# Ada-DF++: A Dual-Branch Adaptive Facial Expression Recognition Method Integrating Global-Aware Spatial Attention and Squeeze-and-Excitation Attention

**DOI:** 10.3390/s25175258

**Published:** 2025-08-24

**Authors:** Zhi-Rui Li, Zheng-Jie Deng, Xi-Yan Li, Wei-Dong Ke, Si-Jian Yan, Jun-Du Zhang, Chang Liu

**Affiliations:** 1 School of Information Science and Technology, Hainan Normal University, Haikou 571127, China; 202313085404006@hainnu.edu.cn (Z.-R.L.); xiyanli2006@163.com (X.-Y.L.); yansijian2023@163.com (S.-J.Y.); 202413085404021@hainnu.edu.cn (J.-D.Z.); 202412083900005@hainnu.edu.cn (C.L.); 2 Hainan eUnited-Tech Technology Co., Ltd., Haikou 570209, China; wd.ke@eunited-tech.com

**Keywords:** adaptive feature fusion, Squeeze-and-Excitation attention, facial expression recognition, Global-Aware Spatial attention

## Abstract

Facial Expression Recognition (FER) is a research topic of great practical significance. However, existing FER methods still face numerous challenges, particularly in the interaction between spatial and global information, the distinction of subtle expression features, and the attention to key facial regions. This paper proposes a lightweight Global-Aware Spatial (GAS) Attention module, designed to improve the accuracy and robustness of FER. This module extracts global semantic information from the image via global average pooling and fuses it with local spatial features extracted by convolution, guiding the model to focus on regions highly relevant to facial expressions (such as the mouth and eyes). This effectively suppresses background noise and enhances the model’s ability to perceive subtle expression variations. In addition, we further introduce a Squeeze-and-Excitation (SE) Attention module into the dual-branch architecture to adaptively adjust the channel-wise weights of features, emphasizing critical region information and enhancing the model’s discriminative capacity. Based on these improvements, we develop the Ada-DF++ network model. Experimental results show that the improved model achieves test accuracies of 89.21%, 66.14%, and 63.75% on the RAF-DB, AffectNet (7cls), and AffectNet (8cls) datasets, respectively, outperforming current state-of-the-art methods across multiple benchmarks and demonstrating the effectiveness of the proposed approach for FER tasks.

## 1. Introduction

In current facial research, facial recognition [1] has become highly mature, while facial emotion analysis [2] is still rapidly developing and holds vast potential for future exploration. In daily life, facial expressions serve as the main and most direct basis for judging a person’s emotions [3]. Facial emotion analysis not only plays a vital role in emotion recognition but also has applications in medical diagnosis [4], education and teaching [5], and assisted driving [6].

In medical diagnosis, a person’s emotional state can be assessed through facial emotion analysis to identify issues such as emotional abnormalities or psychological depression [4]. For example, during sessions with a psychologist, facial emotion detection can help determine whether a person may be suffering from depression, anxiety, or other psychological disorders. It can also be used to infer physical discomfort resulting from irregular lifestyles.

In educational settings, facial analysis can be used to evaluate students’ learning states or attentiveness during classes [5]. For instance, in a classroom environment, surveillance cameras can monitor changes in students’ facial expressions in real time to analyze their learning status, such as looking down, being distracted, or concentrating. This approach is also applicable to online education [7], where students’ emotional states—such as anxiety, confusion, seriousness, or relaxation—can be monitored through webcams. Timely interventions can then be made to help adjust their learning state and improve efficiency.

In assisted driving, facial expression analysis can help evaluate the driver’s status [6]. For instance, repeated drowsiness or eye closure may indicate that the driver is in a fatigued state, while signs of anger or frequent looking around may indicate emotional instability. Such detections can trigger preventive measures to avoid accidents.

In recent years, many studies on facial emotion recognition have been conducted in indoor settings, such as laboratories, computer rooms, or classrooms [8]. Others have collected data from real-world environments, such as outdoor sports or fieldwork [9]. Common emotion categories include happiness, contempt, anger, surprise, sadness, neutrality, fear, and disgust.

Although facial emotion analysis has been widely applied in various fields, it still faces multiple challenges in real-world scenarios, such as subtle differences between expressions, large variations in lighting and head pose, complex background interference, and inconsistent annotation standards across different datasets. These issues can significantly reduce the model’s generalization ability and recognition accuracy, making robust facial emotion analysis a critical and challenging task.

The FER2013 dataset, first introduced by Goodfellow et al. [10] during the 2013 Kaggle competition “Challenges in Representation Learning: Facial Expression Recognition,” contains seven emotion categories: happiness, anger, surprise, sadness, neutral, fear, and disgust. The AffectNet dataset, proposed by Mollahosseini et al. [11], is the largest and most diverse in-the-wild facial expression dataset to date, containing eight categories: happiness, contempt, anger, surprise, sadness, neutral, fear, and disgust.

The RAF-DB dataset, introduced by Li et al. [12], has two versions: the first version contains seven categories similar to FER2013, while the second version includes eleven compound emotion categories such as happy-surprised, happy-disgusted, sad-fearful, sad-disgusted, surprised, neutral-disgusted, disgusted-surprised, fearful neutral-happy, neutral-sad, neutral-angry, and neutral-fearful.

In addition to the static image datasets mentioned above, dynamic datasets can also be used for facial analysis. For instance, the DAiSEE dataset proposed by Abedi et al. [8] in 2018 was recorded in real classrooms or online learning environments. It includes indoor scenes, various lighting conditions, and different facial angles. The dataset includes four categories—engagement, excitement, boredom, and frustration—each divided into four intensity levels, with every video lasting 10 s.

The Engagement dataset, proposed by Dhall and Singh et al. [13,14], also consists of 10-second videos mainly collected from real classroom scenarios. It simulates daily learning environments and includes various learning tasks and diverse emotional expressions, with participation levels classified into low, moderate, high, and full engagement.

In the study of facial expression datasets, one of the mainstream methods is the Adaptive Label Distribution Fusion Network (Ada-DF) [15]. This network is built upon a ResNet-18 [16] backbone pre-trained on the MS-Celeb-1M [17] dataset. While maintaining low computational cost, it effectively alleviates the problem of label ambiguity by learning the distribution of sample labels, thereby improving the model’s adaptability to uncertain annotations. Additionally, this framework constructs an auxiliary branch to compute label distribution and integrates information adaptively through an attention mechanism, optimizing the training of the target branch and achieving good performance on FER tasks.

However, the Ada-DF framework fails to effectively co-model spatial information and local details during feature extraction, resulting in limited discrimination ability between expression categories, especially when handling similar expressions. Moreover, the model does not sufficiently focus on key facial regions (such as the eyes, eyebrows, and mouth corners), making it vulnerable to background noise, which affects classification accuracy.

To address these issues, we revisited the design of the Ada-DF network and proposed an improved version named Ada-DF++.The overall objective of Ada-DF++ is to simultaneously enhance global and local feature representation, improve robustness to complex backgrounds, and increase the model’s ability to distinguish subtle and fine-grained expressions in facial emotion recognition scenarios.

In this work, we restructured the ResNet-18 architecture and introduced the GAS attention mechanism, which preserves spatial structure while fusing contextual features. It can adaptively learn the salient local regions associated with different expression categories, enhancing inter-class separability and intra-class compactness, thereby more accurately capturing subtle expression features with spatial dependencies. Meanwhile, we replaced the traditional attention mechanism in the dual branches of the ResNet-18 with the SE attention mechanism [18], which addresses the insufficient focus on key facial areas.

Combining the above improvements, we proposed a more comprehensive Ada-DF++ network model. In summary, the contributions of this paper are as follows:

1. This paper proposes a Global-Aware Spatial Attention (GAS Attention) module, which introduces a semantic-aware mechanism based on global average pooling to adaptively fuse global semantic information with local spatial features. This module effectively identifies key regions corresponding to different facial expression categories. It not only enhances inter-class separability and intra-class compactness but also improves the model’s ability to perceive subtle expression differences, thereby significantly boosting its representation of local spatial information and overall recognition performance.
2. This paper replaces the traditional attention mechanism in the branch with the Squeeze-and-Excitation Attention (SE Attention) module, which dynamically adjusts channel weights of key regions. This enhances the model’s focus while suppressing irrelevant features, thereby reducing misclassifications. Additionally, it improves generalization performance and helps mitigate overfitting.
3. In this study, Ada-DF++ was evaluated on the AffectNet-7, AffectNet-8, and RAF-DB datasets, achieving accuracy rates of 66.14%, 63.75%, and 89.21%, respectively. Compared with the Ada-DF [15] model, these results represent improvements of 1.57%, 1.75%, and 1.11%. Ada-DF++ outperforms the current mainstream network models.

## 2. Related Works

In recent years, facial emotion recognition (FER) has become a hot research topic in the field of human–computer interaction. Jie et al. [19] improved semi-supervised facial expression recognition using an unlabeled face recognition dataset. Additionally, they proposed a hybrid data augmentation strategy for facial images and determined the loss weight of real images based on the intersection and union (IoU) of faces in two images. Fei et al. [20] proposed a global multi-scale extraction and local hybrid multi-head attention network (GLMEA) to address accuracy issues caused by factors such as face occlusion and head pose variations. Xu et al. [21] introduced a multi-scale facial expression recognition model based on dynamic global and static local attention (MFER), which captures the differences in category features in FER tasks and improves inter-class separability and intra-class compactness. Savchenko et al. [22] proposed a pipeline algorithm based on video facial processing. First, they used face detection, tracking, and clustering techniques to extract each student’s facial sequence. Then, a single effective neural network was used to extract emotion features from each frame. Zheng et al. [23] proposed a dual-stream pyramid cross-fusion Transformer network (POSTER) to solve the problems of inter-class similarity, intra-class differences, and scale sensitivity. Hsu et al. [24] proposed a new three-attribute perceptron network (TAPNet), which effectively utilizes global, local, and key sub-regions of facial features to fully exploit the diverse potential information provided by each facial attribute. Liu et al. [15] tackled the FER task through a label distribution learning paradigm and developed a dual-branch adaptive distribution fusion (Ada-DF) framework.

Since the foundation laid by the classic convolutional neural network LeNet-5 [25] in 1998, the structure of CNNs has continuously evolved, providing significant assistance in the development of computer vision. In 2012, Krizhevsky et al. [26] introduced AlexNet, which adopted a five-layer convolutional and three-layer fully connected structure and used the ReLU activation function instead of the Sigmoid activation function to accelerate computation and reduce the vanishing gradient problem. Later, VGGNet [27] used 3 × 3 small convolutional kernels instead of larger kernels, reducing the number of parameters while enhancing non-linear expression capabilities. ResNet [28] introduced residual connections to address the vanishing gradient problem in deep neural networks. Today, Vision Transformer [29] has introduced a self-attention mechanism, which enables CNNs to break through limitations and become a mainstream technical framework. However, Vision Transformer has higher computational complexity and requires a larger GPU scale. In contrast, lightweight ResNet, with much smaller computational costs than Vision Transformer, performs better in classification tasks on smaller image datasets.

The core idea of ResNet is residual learning. It decomposes the network mapping H(x) into a residual learning function F(x) = H(x) − x, which is then reconstructed as H(x) = F(x) + x, enabling direct learning of the difference between the input and the desired output, thereby alleviating the vanishing gradient problem. A residual block consists of three convolutional layers, BatchNorm (BN), and the ReLU activation function. The input xxx of the first layer passes through a residual block to obtain F(x), which is then added to x via a skip connection to obtain H(x). In deeper ResNet architectures, such as ResNet-50, ResNet-101, and ResNet-152, 1 × 1 convolutions are used for dimensionality reduction, followed by 3 × 3 convolutions to extract features, and 1 × 1 convolutions to restore the dimensions. This reduces computational complexity, decreases the number of parameters, and maintains sufficient feature expression capability.

In facial emotion analysis, the differences in facial expressions are subtle, and many studies incorporate attention mechanisms during experimentation. The spatial attention mechanism proposed by Woo et al. [30] pays more attention to key facial features, such as the eyes, nose, and mouth, to enhance the acquisition of critical information while reducing background interference. However, it lacks global modeling ability and may lose key information. Ashish et al. [31] formally introduced the self-attention mechanism in the Transformer paper for natural language processing, which was later applied to computer vision. The self-attention mechanism can compute the global relationships within sequences or different regions of an image, capturing global information. However, its computational complexity is high, and it lacks focus on local features. Xue et al. [32] proposed Patch and Token pooling attention mechanisms. The Patch attention mechanism divides an image into multiple patches, then applies Transformer computation to each patch, while the Token pooling mechanism gradually reduces the number of tokens during multi-layer Transformer computations to reduce computational load while extracting important information. Additionally, Ma et al. [33] proposed an attention-based selective fusion mechanism to encode multiple global-local features, but this mechanism performed poorly in FER tasks. Liu et al. [34] introduced a method that includes a confidence estimation module and a weighted regularization module to address uncertainty in facial expression recognition. Xue et al. [35] emphasized the importance of local features in expression recognition and designed a multi-attention dropout module to mine local features from different regions. Although these methods have achieved good performance in FER tasks, they failed to fully leverage the contextual information of facial expressions and did not effectively facilitate collaborative learning between spatial and global information. To address these issues, we designed a GAS Attention. This mechanism extracts local spatial features through convolution operations and enhances feature expression capability by combining BatchNorm2d and non-linear activation functions. It retains spatial structure information while guiding the model to focus on key regions closely related to emotional changes. Especially when responding to subtle expression changes, GAS Attention effectively integrates local and global semantic information, enhancing the model’s ability to perceive and discriminate emotional features, thus enabling more precise emotional analysis.

## 3. Materials and Methods

In this section, we first review the model process of Ada-DF, followed by a description of the Ada-DF++ framework. We then discuss the specific details of the dual-branch structure, the global spatial-aware attention mechanism in the backbone, and the channel attention mechanism in the branches.

### 3.1. Brief Review of Ada-DF

The Ada-DF framework is built upon a ResNet18 [17] backbone pretrained on MS-Celeb-1M [16] and consists of a frozen feature extractor, two high-level convolutional branches, two attention modules, and two fully connected classifiers. The feature extractor is used to stably extract facial features. Among the two branches, the auxiliary branch is responsible for generating label distributions and class distributions of emotions, while the target branch is used for final prediction. Attention modules are embedded in the last layers of both branches to determine whether a sample is “certain,” producing corresponding attention weights w_aux_ and w_tar_, whose average w_avg_ is used for the subsequent distribution fusion.

Rank regularization is applied to enhance the contrast in attention weights and normalize them to the range w_min_, where w_min_ is set to 0.2 based on empirical results.

During class distribution mining, the auxiliary branch outputs label distributions for each sample, from which class-level distributions are derived. To handle instability in the early training phase of the auxiliary branch, a fixed “threshold distribution” is used to replace class distributions that do not meet a preset threshold t. The auxiliary branch is trained with cross-entropy loss.

Due to biases in label distributions and intra-class variation, an adaptive distribution fusion mechanism is proposed. It fuses the label and class distributions based on attention weights, producing final fused distributions to supervise the training of the target branch. Samples with higher attention weights rely more on class distributions, while those with lower weights depend more on the auxiliary branch’s label predictions. The target branch is trained using the KL divergence between the fused and predicted distributions. The total loss combines KL loss, cross-entropy loss, and rank regularization with dynamic weighting functions based on training epochs, the auxiliary branch is emphasized during the early epochs to obtain reliable distributions, and the target branch becomes the focus in later stages to prevent overfitting, thereby achieving overall performance optimization.

### 3.2. Overall Architecture

**Figure 1:**
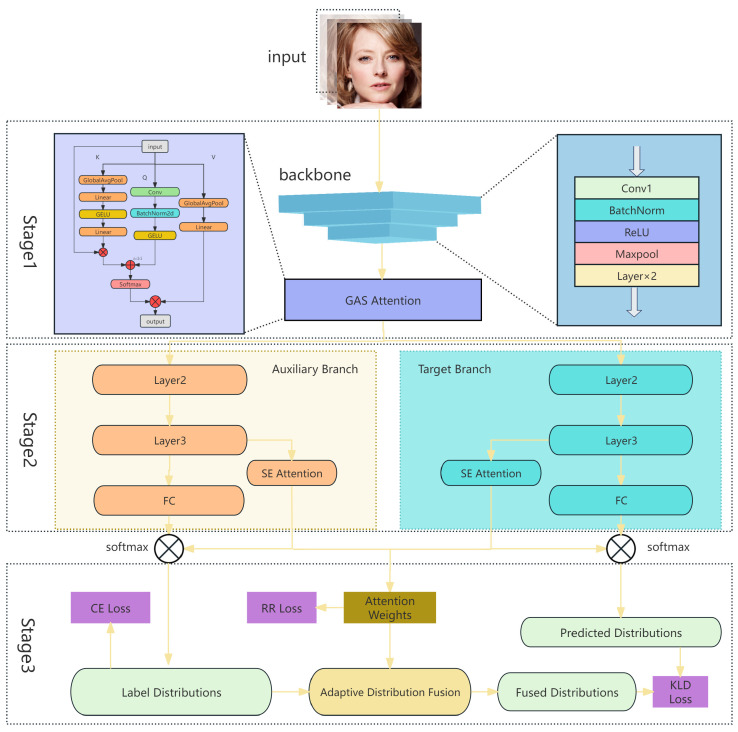
The input facial images are first processed in Stage 1, where ResNet18 is used to extract shared features, which are then enhanced through the GAS Attention module. The enhanced features are fed into Stage 2, a dual-branch network consisting of an Auxiliary Branch and a Target Branch, both incorporating the SE attention mechanism to improve channel-wise feature representation. Finally, in Stage 3, the outputs of the two branches are integrated through adaptive distribution fusion with attention weights, producing the final prediction distribution.

Based on the Ada-DF model described in Section 3.1, we construct an improved framework that integrates dual attention mechanisms to enhance the model’s ability to capture both local and global features in facial expressions, as show in Figure 1. Specifically, the model first employs the frozen first five layers of ResNet18 as a shared feature extractor to encode input facial images into intermediate feature maps. The GAS Attention module, which contains 206.15 K trainable parameters, is applied to the feature map output from the frozen layers of ResNet-18. It extracts semantic-level Key and Value information through global average pooling and generates the Query using a 7 × 7 convolution, which enlarges the receptive field and emphasizes key facial regions such as the corners of the eyes and mouth. After fusion, GELU activation, BatchNorm, and Softmax are applied to construct a spatial attention map that effectively highlights expression-relevant regions (such as the eyes and mouth corners) and suppresses background noise, improving the perception of subtle expressions.

The enhanced feature map is then fed in parallel into two structurally identical sub-branches: the Auxiliary Branch and the Target Branch. The Auxiliary Branch generates label distributions for each sample and derives class-level distributions, providing prior knowledge for uncertain samples. A fixed threshold distribution is used early in training for unstable predictions. The Target Branch performs the final prediction and is supervised by the fused distribution of auxiliary predictions and class-level information, weighted adaptively according to attention scores. Each branch contains the Layer2 and Layer3 modules of ResNet18 and further integrates an SE Attention to recalibrate feature responses across channels, enhancing the focus on critical semantic features.

During training, attention weights from both branches, which guides the adaptive distribution fusion. Samples with higher attention rely more on class-level distributions, while lower-weighted samples rely more on the auxiliary branch’s label predictions. Dynamic weighting between auxiliary and target branch losses is applied: early epochs emphasize auxiliary outputs for reliable distributions, whereas later epochs focus on target branch predictions to reduce overfitting.

### 3.3. GAS Attention Model

In facial expression recognition, there is often a high degree of similarity in facial regions across different emotional categories, which makes it challenging for models to distinguish them accurately. For instance, the nose region shows minimal variation across various emotional states—even during anger, when slight muscle twitching may occur, it is often difficult for traditional attention mechanisms to effectively capture such subtle changes. If these fine-grained expression differences cannot be modeled, the model’s ability to discriminate between emotions is greatly compromised. Therefore, enhancing the model’s sensitivity to subtle expression differences is key to improving emotion recognition performance.

**Figure 2:**
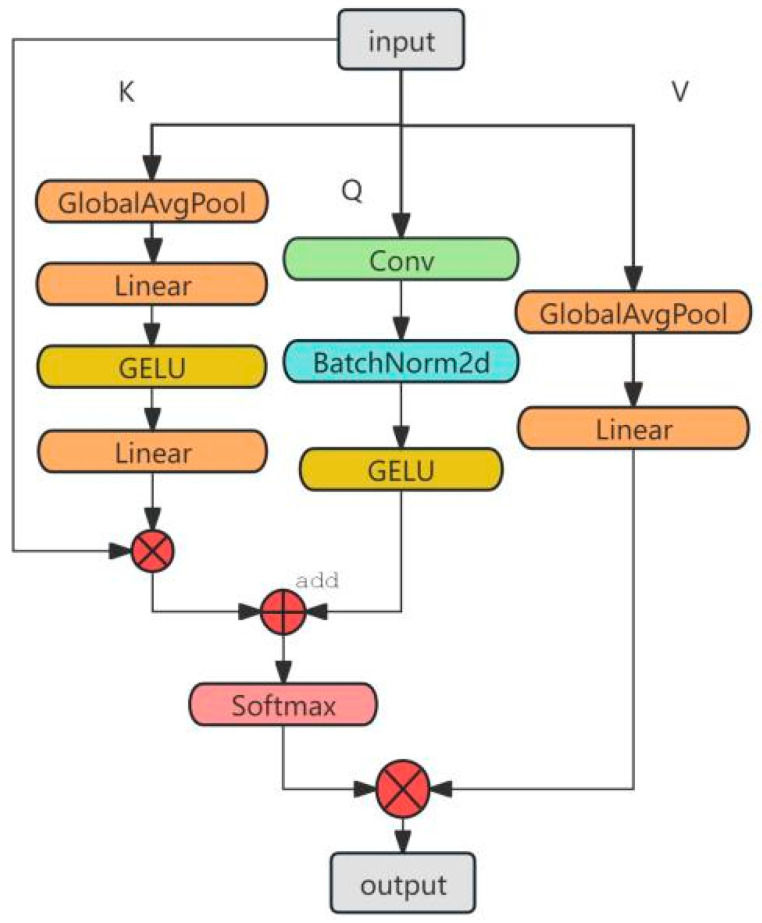
Architecture of GAS attention.

To address this, inspired by CoTNet and self-attention mechanisms, we designed a GAS Attention, as illustrated in Figure 2. This mechanism first applies global average pooling to the input feature map to extract the Key and Value representations, capturing global semantic features. Simultaneously, the input feature map is passed through a convolution layer to obtain local Query representations, which are further enhanced via BatchNorm2d and GELU activation to strengthen the expression of local features. The Query and Key are then fused through weighted interaction, and a spatial attention map is generated via Softmax, achieving semantic interaction between global and local features.

This attention map is then applied to the Value, emphasizing the responses in key facial regions—such as the eye corners and mouth corners—where micro-expressions are rich, thereby increasing the model’s sensitivity to subtle emotional changes. The introduction of the GELU activation function provides a smoother non-linear response and enhances consistency in the feature space, allowing the model to learn clearer class boundaries in high-dimensional feature spaces.

Compared with traditional spatial attention mechanisms, the GAS Attention establishes a connection between spatial information and global contextual semantics, significantly improving the network’s capacity for both structural and semantic modeling. Although the backbone network already possesses certain semantic feature extraction capabilities, introducing the GAS Attention in the deep feature fusion stage helps alleviate the disconnection among deep semantic representations, ultimately enhancing overall facial expression recognition performance.

Compared with the traditional self-attention mechanism, which relies on simple linear mappings to construct attention features, the GAS Attention combines global awareness with local spatial modeling capabilities. By using global average pooling and local convolution operations, it effectively enhances the ability to model both spatial structure and semantic context. Specifically, the input feature X first undergoes a 7 × 7 convolution operation to extract local spatial features, which are then normalized by BatchNorm2d and passed through the GELU activation function to obtain local feature maps. Meanwhile, the input feature X undergoes global average pooling (Global Average Pooling) to extract channel-level global semantic information, which is then fed into the Key and Value branches. The Key branch constructs the structure-aware attention vector K through two layers of linear transformations and GELU activation, while the Value branch generates the value vector V through a linear transformation, used to adjust the importance of different positions.

Next, the local feature Q is added to the global structure-aware vector K, and the Softmax function is applied to generate an attention weight matrix, which is used to weight the Value vector V. The final attention-enhanced output is then obtained. Finally, this enhanced output is added to the input residual to improve the model’s expressive capability. This mechanism introduces global semantic guidance during implementation, enabling attention to focus more accurately on key regions, which is particularly useful for tasks like facial expression recognition, where small differences need to be modeled.

It improves inter-class separability and intra-class compactness.

(1) $$X_{pooled}=GAP(X)$$

(2) $$Q=GELU(BN(Conv_{7\times 7}(X_{pooled\_upshape})))\epsilon R^{C\cdot H\cdot W}$$

(3) $$X^{\prime }=GELU(linear_{1}(X_{pooled})\epsilon R^{C}$$

(4) $$K=GELU(Linear_{1}(Linear_{0}(X_{pooled})))\times X_{pooled}$$

(5) $$K^{\prime }=K_{upshape}⊗X\epsilon R^{C\cdot H\cdot W}$$

(6) $$V=Linear(X_{pooled\_permute})\epsilon R^{C}$$

where $Linear_{0}$ is the first linear transformation, $linear_{1}$ is the second linear transformation, ⊗ represents the Hadamard product operation, and $X_{pooled\_permute}$ is the result of $X_{pooled}$ after dimension transformation.

In addition, considering that convolution operations can effectively extract local features while maintaining spatial structure information, convolution layers are used to process the input feature X, combined with BatchNorm normalization and GELU activation functions to extract static local features Q, thereby constructing a global static information matrix. The smooth non-linear properties of the GELU function enhance the feature space’s expressive power, allowing the model to learn more consistent high-dimensional feature representations, thereby improving its ability to distinguish between facial expression categories. Therefore, the GAS Attention, built on the GELU activation function, can not only capture global information of the input image but also effectively integrate spatial information, enhancing the model’s ability to perceive subtle changes in facial expressions.

(7) $$A=Softmax(K^{\prime }+Q)\epsilon R^{C\cdot H\cdot W}$$

The multiplication operation in self-attention helps represent the similar features between $K^{\prime }$ and Q, but it is challenging to express both global perception ability and local modeling ability simultaneously. To address this, we use addition operations to ensure that the attention matrix A contains both types of information, thereby providing a more comprehensive representation of the input features.

Specifically, the attention matrix A is learned jointly from the static local information matrix Q and the dynamic global information matrix K, rather than relying solely on Q and K. This approach effectively integrates contextual dynamic information with static local information. Similar to the self-attention mechanism, we aggregate A and V to obtain the feature information matrix and compute the features that the model focuses on, thereby enhancing the representational capacity of the input feature map.

(8) $$Attention(X)=A⊗V{\epsilon R}^{C\cdot H\cdot W}$$

The GAS Attention introduces global semantic guidance during its implementation, allowing the attention to more accurately focus on key areas. This is particularly useful in facial emotion analysis, where it handles the modeling of subtle differences, thereby enhancing inter-class separability and intra-class compactness.

### 3.4. SE Attention Model

**Figure 3:**
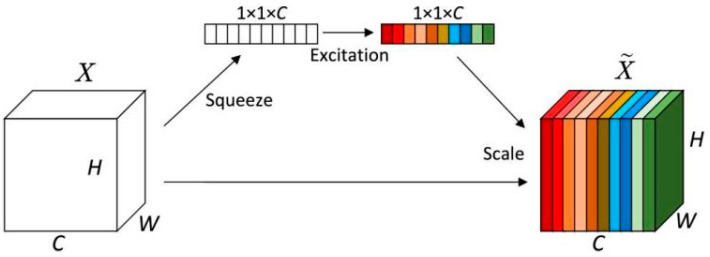
Architecture of SE attention.

In both the auxiliary branch and the target branch, we introduce the SE Attention. The SE Attention adaptively adjusts the weight of each channel by first performing Squeeze and then Excitation, enabling the model to focus on useful features while suppressing irrelevant or redundant information. Applying SE Attention to both branches ensures that they possess comparable feature selection capacity, avoiding an imbalance that could bias the fusion stage. Moreover, using independent SE modules allows each branch to learn branch-specific channel importance patterns, enhancing feature diversity and complementarity. The structure diagram of the SE Attention is shown in Figure 3.

Specifically, the SE Attention consists of three main operations: Squeeze, Excitation, and Scale. The Squeeze operation compresses the C × H × W feature map X into a 1 × 1 × C vector so that each channel can be represented by a single value. After the pooling operation, the feature and vector representation are denoted as ∈, and the operation can be expressed as

(9) $$Z=Squeeze(X)=\frac{1}{H\times W}\sum_{i=1}^{H}\sum_{j=1}^{W}X(i,j)$$

The Excitation operation uses two fully connected (FC) layers and non-linear activation functions (ReLU, Sigmoid) to learn the weights of each channel, capturing the relationships between channels. The calculation formula can be expressed as

(10) $$S=Excitation(Z)=\sigma (W_{2}\delta (W_{1}Z))$$

where $W_{1}\epsilon R^{\frac{c}{r}\times c}$ and $W_{2}\epsilon R^{c\times \frac{c}{r}}$ are two fully connected (FC) layers, δ represents the ReLU activation function, and σ represents the Sigmoid activation function, used to normalize the weights.

The Scale operation uses the weight vector S obtained from Excitation to weight the feature map X, thus generating the desired feature map $\tilde{X}$. It is important to note that the size $\tilde{X}$ of the feature map remains consistent with the original feature map X, so the SE Attention does not change the size of the feature map. The expression for Scale is as follows:

(11) $$\tilde{X}=Scale(X,S)=XS$$

In summary, the SE Attention adaptively adjusts the weights of each channel, effectively enhancing the model’s focus on key features while suppressing irrelevant or redundant information. In facial image emotion analysis tasks, the SE Attention first compresses the input feature map into a channel description vector through the Squeeze operation. Then, during the Excitation phase, it learns the inter-channel dependencies using fully connected layers and non-linear activation functions. Finally, the Scale operation applies the learned weights to the feature map, highlighting emotion-related features. This mechanism strengthens the expression of key facial regions while maintaining the original spatial dimensions of the feature map, improving sensitivity to subtle facial expression changes. By introducing the SE Attention in both the auxiliary and target branches, the model can optimize the information flow during multi-branch feature fusion, further improving the accuracy and robustness of emotion classification.

## 4. Experiments

We validated the effectiveness of Ada-DF++ on three standard FER public datasets: RAF-DB, AffectNet (7cls), and AffectNet (8cls). Below, we present detailed experimental settings and some of the results. First, we compare Ada-DF with several state-of-the-art methods. Then, we conduct extensive model analysis and ablation studies on Ada-DF++.

### 4.1. Experimental Details

Datasets. We evaluate the FER performance of Ada-DF++ on three widely used public datasets: RAF-DB, AffectNet (7cls), and AffectNet (8cls). RAF-DB is a large-scale facial expression database annotated by 315 individuals (students and university staff). For expression selection, RAF-DB chose seven expressions (six basic emotions and a neutral emotion) from a variety of options, such as smile, giggle, cry, anger, fear, fright, shock, surprise, disgust, and neutral. It mainly contains 12,271 training images and 3068 testing images. AffectNet is currently the largest public dataset in the FER field. It contains approximately 1 million facial images associated with emotional keywords. It mainly includes eight basic emotions (neutral, happy, angry, sad, fear, surprise, disgust, and contempt). We primarily use the seven-class (excluding contempt) and eight-class versions of the AffectNet dataset. AffectNet (7cls) consists of 280,401 training images and 3500 validation images (500 images per category). AffectNet (8cls) consists of 283,501 training images and 4000 validation images (500 images per category). The specific dataset statistics are shown in Table 1.

Settings. Before training, all image pixels were resized to 100 × 100, and all images in the FER datasets were preprocessed with MTCNN for face detection and alignment. RandAugment, random erasing, and random horizontal flipping were applied for data augmentation. For training on the RAF-DB dataset, the optimal performance was achieved with the parameters epoch = 75, batch_size = 64, threshold = 0.7, and beta = 3. For the AffectNet (7cls) and AffectNet (8cls) datasets, the best performance was obtained with epoch = 75, batch_size = 64, threshold = 0.5, and beta = 5. Detailed experimental results can be found in Section 4.3. Next, we describe the detailed experimental process and results.

### 4.2. Comparative Experiments

To validate the effectiveness of the proposed method in facial expression recognition tasks, we conducted performance comparisons with state-of-the-art methods on three benchmark datasets: RAF-DB, AffectNet-7-class subset (AffectNet (7cls)), and AffectNet-8-class subset (AffectNet (8cls)), as shown in Table 2. The table lists the test accuracies of various advanced methods, including SCN, DACL, VTFF, EfficientFace, MA-Net, LDL-ALSG, RAN, KTN, PAT, and IPD-FER, and compares them with our model, Ada-DF++.

As observed from the results in Table 2, our method achieved an accuracy of 89.21% on the RAF-DB dataset, significantly outperforming other methods. Models with comparable performance, such as IPD-FER and MA-Net, achieved 88.89% and 88.76%, respectively, still slightly lower than our method, demonstrating the generalizability and effectiveness of our proposed modules across multiple datasets.

On the more challenging AffectNet-7 and AffectNet-8 datasets, our method also achieved leading performance, with accuracies of 66.14% and 63.75%, respectively. In comparison, traditional efficient networks like EfficientNet-B0 and B2 achieved 60.8%/57.55% and 64.3%/63.03%, while mainstream methods such as DACL, KTN, and MA-Net also fell short of our model’s performance. This further confirms the superior adaptability and robustness of our method across different expression categories.

Additionally, compared with the Ada-DF model (without the GAS Attention module and SE Attention module, our method achieved improvements of 1.11%, 1.57%, and 1.75% on the three datasets, respectively. This highlights the significant advantage of the proposed GAS Attention and the introduction of the SE Attention in extracting effective features and enhancing the model’s discriminative power.

### 4.3. Ablation Study

To evaluate the impact of GAS Attention and SE Attention on model performance, we conducted extensive experiments to analyze the influence of different modules. The final results are shown in the table below. Without incorporating GAS Attention and SE Attention, the accuracy on the three FER datasets, RAF-DB, AffectNet (7cls), and AffectNet (8cls), was 88.10%, 64.57%, and 62.20%, respectively. When only GAS Attention was introduced, the accuracy increased to 88.66%, 65.29%, and 63.00%, representing improvements of 0.56%, 0.72%, and 1.20%, respectively. When only SE Attention was introduced, the accuracy rose to 88.49%, 65.40%, and 62.62%, with gains of 0.39%, 0.83%, and 0.42%, respectively. When both LS Attention and SE Attention were applied simultaneously, the accuracy further increased to 89.21%, 66.14%, and 63.75%, showing improvements of 1.11%, 1.57%, and 1.55%, respectively. The results are visually summarized in Table 3.

In the experimental design, two key control parameters were introduced to improve the reliability of the auxiliary branch label distribution and the overall robustness of the model: confidence threshold t and delayed fusion threshold β.

**Threshold t**: During the auxiliary branch label distribution process, some predictions may be incorrect or of low quality. Therefore, parameter t is introduced to filter out unreliable samples. A sample is only considered reliable if the predicted probability from the auxiliary branch exceeds the threshold t. By setting threshold t, high-confidence samples can be selected to construct the class distribution, thereby improving robustness and accuracy.

**Threshold β**: In the early stages of training, the auxiliary branch label distribution is unreliable, so an accurate class distribution cannot be constructed immediately. To address this, a delayed mechanism with threshold β is introduced. For the first β epochs, class distribution is not used for fusion; only label distribution is considered. It is only after β + 1 epochs that class distribution and attention-weighted fusion are introduced.

Experiments were conducted on the RAF-DB and AffectNet datasets to evaluate different settings for t and β. The results show that for the RAF-DB dataset, the best performance was achieved with t = 0.7 and β = 3, reaching an accuracy of 89.21%. For AffectNet (7cls) and AffectNet (8cls), the highest accuracies of 66.14% and 63.75%, respectively, were obtained with t = 0.5 and β = 5. These results indicate that reasonable settings for threshold t and delayed mechanism β can effectively enhance the model’s tolerance to uncertain samples and improve the adaptability and accuracy of the fusion strategy in diverse data scenarios. Detailed experimental results are shown in Table 4 and Table 5.

### 4.4. Analysis of Different Attention Mechanisms

To rigorously evaluate the effectiveness of the proposed attention mechanism, we incorporated three representative modules into the baseline model for comparative experiments, namely CBAM, ECA, and the proposed GAS, and assessed their performance on the RAF-DB and AffectNet datasets. CBAM and ECA were selected as baselines because they represent two typical paradigms of attention design. Specifically, CBAM enhances feature representation by combining channel and spatial attention and has been widely used in image analysis tasks, making it a standard reference method in facial expression recognition. In contrast, ECA models channel dependencies using one-dimensional convolution, avoiding redundant fully connected operations, which reflects a trend toward lightweight attention design.

Experimental results indicate that, although CBAM can enhance feature representation by emphasizing salient regions, its spatial attention is easily affected by background and irrelevant areas, limiting the model’s ability to capture fine-grained facial details, especially under multi-class and complex scenarios. ECA demonstrates certain advantages in modeling channel dependencies, but it mainly focuses on local relationships and lacks global dependency modeling, which hampers its ability to capture long-range interactions and limits performance in fine-grained classification. In contrast, the proposed Global Attention with Spatial awareness (GAS) explicitly incorporates global dependencies during feature modeling, enabling the model to capture both overall facial structures and local discriminative details. As shown in Table 6, GAS achieves the best performance on RAF-DB, AffectNet (7cls), and AffectNet (8cls). Notably, on AffectNet (7cls), GAS outperforms CBAM by 1.28% and ECA by 2.71%. These results demonstrate that GAS consistently provides performance advantages across different datasets and classification granularities, reflecting strong robustness and generalization capability.

### 4.5. Sensitivity Analysis

Table 7 presents the impact of different kernel sizes in the GAS attention mechanism on model computational cost (Model FLOPs), GAS module FLOPs (GAS FLOPs), model parameters (Model Params), GAS module parameters (GAS Params), and classification accuracy (Acc%). It can be observed that as the kernel size increases from 3 × 3 to 9 × 9, both the FLOPs and parameters of the GAS module grow significantly. For instance, on the RAF-DB dataset, GAS FLOPs increase from 29.16 M to 260.37 M, and GAS Params rise from 42.31 K to 337.22 K, while the overall model FLOPs also increase but the model parameters change only slightly. This indicates that larger kernels substantially increase the computational cost of the GAS module while having a limited effect on the overall model size.

In terms of classification accuracy, on the RAF-DB dataset, accuracy improves from 88.23% to 89.21% when the kernel size increases from 3 × 3 to 7 × 7 but slightly drops to 88.23% with a 9 × 9 kernel. This suggests that overly large kernels may introduce redundant features and negatively affect performance. On the AffectNet datasets (seven-class and eight-class), accuracy slightly improves with larger kernels but shows no further gain or even a slight decline at 9 × 9. These results indicate that a medium-sized kernel, such as 7 × 7, can capture global contextual information effectively while keeping computational cost manageable.

Overall, using a 7 × 7 kernel in the GAS attention mechanism achieves high accuracy across datasets, with reasonable computational cost and parameter size, providing a good balance between performance and efficiency. Our experiments confirm the initial hypothesis that a moderate kernel size is more suitable for capturing expressive emotional features, whereas kernels that are too small or too large may either limit feature representation or increase computational burden.

**Figure 4:**
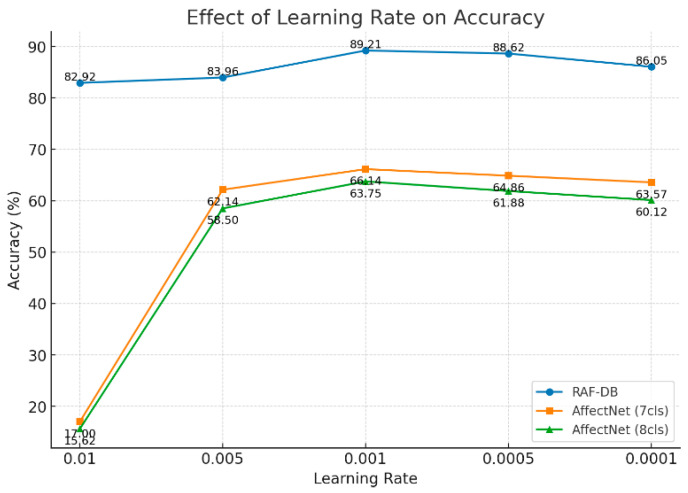
This figure presents a comparison of the model’s classification accuracy on the RAF-DB, AffectNet (7cls), and AffectNet (8cls) datasets under different learning rate settings.

At the same time, this study also conducted experiments to analyze the sensitivity of the model to different learning rates, as shown in Figure 4. In these experiments, the number of training epochs was fixed at 100, and the classification performance of the model on the RAF-DB and AffectNet datasets under different learning rates was compared. The results indicate that the learning rate has a significant impact on model performance.

On the RAF-DB dataset, the model achieved the highest accuracy of 89.21% when the learning rate was 0.001, significantly outperforming other settings. A too-large learning rate (0.01) caused unstable training, reducing the accuracy to 82.92%, while a too-small learning rate (0.0001) slowed convergence, resulting in an accuracy of only 86.05%.

On the AffectNet dataset (7-cls and 8-cls), an excessively large learning rate led to extremely low accuracy of 17.00% and 15.62%, indicating that the model could hardly converge effectively. As the learning rate decreased to 0.001, accuracy increased rapidly, reaching peak values of 66.14% and 63.75%, while further reductions in the learning rate caused a slight drop in performance.

Overall, a moderate learning rate (0.001) is most suitable for training the model on all three datasets. Both excessively large and small learning rates can affect model convergence and final performance, and the results also reflect that the AffectNet dataset, with its larger sample size and more complex categories, is more sensitive to the learning rate.

### 4.6. Performance with Standard Deviation and Statistical Significance

Table 8 presents a comparison between the baseline model (Ada-DF) and our improved method (Ada-DF++) on the RAF-DB and AffectNet datasets. The results are reported as mean ± standard deviation (mean ± std) over multiple runs.

As shown in the table, our method outperforms the baseline across all datasets and classification settings. Specifically, on RAF-DB, our method achieves an average accuracy of 89.21%, representing an improvement of 1.11% over the baseline. On the AffectNet dataset, the improvements are 1.57% and 1.75% for the seven-class and eight-class tasks, respectively.

The standard deviations are within an acceptable range, indicating that both the baseline and our method are generally stable across 10 runs. In the RAF-DB and AffectNet (8-cls) datasets, the standard deviation of our method is slightly higher than that of the baseline, which is reasonable and expected, as more complex models may exhibit minor fluctuations during training. Nevertheless, the consistent improvement in mean accuracy demonstrates that our method enhances performance while maintaining training stability.

In summary, the experimental results validate that Ada-DF++ achieves better average performance than Ada-DF, and the small standard deviations further reflect the reliability and stability of our method.

## 5. Conclusions

This paper addresses key limitations in current facial expression recognition (FER) methods, particularly the insufficient integration of global and local information, poor discrimination of subtle expressions, and inadequate attention to critical facial regions. To overcome these challenges, we propose an enhanced Ada-DF++ model by incorporating a GAS Attention module and an SE Attention module within a dual-branch architecture. The GAS Attention module guides the network to focus on expression-relevant spatial regions by fusing global semantics with local features, while the SE Attention module adaptively recalibrates channel-wise feature responses, further emphasizing critical expression cues. Together, these modules enhance the network’s ability to suppress irrelevant information and highlight subtle yet significant features. Experimental results across multiple benchmark FER datasets confirm the model’s superior accuracy and robustness compared with existing methods. Future research will aim to further advance attention mechanisms to better address complex and dynamic expression patterns, while also exploring their potential in multimodal emotion recognition tasks, with testing and validation in real-world contexts such as healthcare, driving, and education.

## Figures and Tables

**Table 1:** Dataset Scale and Category Distribution Used for Ada-DF++ Model Evaluation (RAF-DB and AffectNet) [36].

Dataset	Train Size	Test Size	Classes
RAF-DB	12,271	3068	7
AffectNet(7cls)	280,401	3500	7
AffectNet(8cls)	283,501	4000	8

**Table 2:** Comparison with the state-of-the-art.

Method	RAF-DB	AffectNet (7cls)	AffectNet (8cls)
SCN [37]	87.03	-	60.23
DACL [38]	87.87	65.20	60.75
VTFF [33]	88.14	61.85	-
EfficientFace [39]	88.36	63.70	59.89
MA-Net [40]	88.42	64.53	60.29
LDL-ALSG [41]	88.53	-	-
RAN [42]	86.9	-	-
MobileNet-v1 [43]	-	60.4	56.88
EfficientNet-B0 [44]	-	60.8	57.55
KTN [45]	88.07	63.97	-
EfficientNet-B2 [46]		64.3	63.03
PAT [47]	88.43	-	-
IPD-FER [48]	88.89	62.23	-
Ada-DF [15]	88.10	64.57	62.00
Ours (Ada-DF++)	89.21	66.14	63.75

**Table 3:** Analysis of the impact of GAS Attention and SE Attention on Ada-DF++ performance across three datasets.

y	GAS	SE	RAF-DB	AffectNet (7cls)	AffectNet (8cls)
√			88.10%	64.57%	62.20%
√	√		88.66%	65.29%	63.00%
√		√	88.49%	65.40%	62.62%
√	√	√	89.21%	66.14%	63.75%

**Table 4:** Impact of different t settings on the performance of the Ada-DF++ model on the FER dataset.

Dataset	t				
	0.4	0.5	0.6	0.7	0.8
RAF-DB	88.04	88.17	88.36	89.21	87.68
AffectNet (7cls)	65.71	66.14	65.70	65.43	64.43
AffectNet (8cls)	63.00	63.75	62.00	61.50	61.12

**Table 5:** Impact of different β settings on the performance of the Ada-DF++ model on the FER dataset.

Dataset	β				
	1	3	5	7	9
RAF-DB	88.04	89.21	88.57	88.37	88.10
AffectNet (7cls)	65.00	65.57	66.14	65.52	64.88
AffectNet (8cls)	61.12	62.25	63.75	62.50	62.12

**Table 6:** Performance of the model on RAF-DB, AffectNet (7cls), and AffectNet (8cls) datasets with CBAM, ECA, and GAS attention mechanisms.

Method	RAF-DB	AffectNet (7cls)	AffectNet (8cls)
CBAM [30]	88.27	64.86	62.75
ECA [49]	88.75	63.43	61.75
GAS(ours)	89.21	66.14	63.75

**Table 7:** Impact of different kernel sizes in the GAS attention mechanism on Model FLOPs, GAS FLOPs, Model Params, GAS Params, and Accuracy across datasets.

Kernel Size	Dataset	Model FLOPs (G)	GAS FLOPs (M)	Model Params (M)	GAS Params (K)	Acc(%)
3 × 3	RAF-DB	3.18	29.16	22.25	42.31	88.23
	AffectNet (7cls)	3.18	29.16	22.25	42.31	65.57
	AffectNet (8cls)	3.18	29.16	22.25	42.31	62.88
5 × 5	RAF-DB	3.39	80.54	22.32	107.85	88.59
	AffectNet (7cls)	3.39	80.54	22.32	107.85	65.86
	AffectNet (8cls)	3.39	80.54	22.32	107.85	63.12
7 × 7	RAF-DB	3.69	157.61	22.41	206.15	89.21
	AffectNet (7cls)	3.69	157.61	22.41	206.15	66.14
	AffectNet (8cls)	3.69	157.61	22.41	206.15	63.75
9 × 9	RAF-DB	4.11	260.37	22.55	337.22	88.23
	AffectNet (7cls)	4.11	260.37	22.55	337.22	66.14
	AffectNet (8cls)	4.11	260.37	22.55	337.22	61.75

**Table 8:** Comparison of baseline and our method on RAF-DB and AffectNet datasets. Results are averaged over multiple runs and reported as mean ± std.

Dataset	Baseline (mean ± std)	Ours (mean ± std)	ΔAcc(%)
RAF-DB	88.10 ± 0.87	89.21 ± 0.89	1.11
AffectNet (7cls)	64.57 ± 0.76	66.14 ± 0.72	1.57
AffectNet (8cls)	62.00 ± 0.73	63.75 ± 0.76	1.75

## Data Availability

All datasets used in this study are publicly available. However, due to the agreement signed with the authors stating that the data shall not be directly distributed to third parties, please visit the official website of the dataset or contact the dataset authors (all datasets have been cited in their respective articles) to obtain the complete dataset.

## Abbreviations

The following abbreviations are used in this manuscript:

FER	Facial Expression Recognition
GAS Attention	Global-Aware Spatial Attention
SE Attention	Squeeze-and-Excitation Attention
Ada-DF	Adaptive Label Distribution Fusion Network

## References

[1] Kaur P., Krishan K., Sharma S.K., Kanchan T. (2020). Facial-recognition algorithms: A literature review. Med. Sci. Law.

[2] Hu Y., Zeng Z., Yin L., Wei X., Zhou X., Huang T.S. (2008). Multi-view facial expression recognition. Proceedings of the 2008 8th IEEE International Conference on Automatic Face & Gesture Recognition.

[3] Gera D., Balasubramanian S., Jami A. (2022). CERN: Compact facial expression recognition net. Pattern Recognit. Lett..

[4] Fu G., Yu Y., Ye J., Zheng Y., Li W., Cui N., Wang Q. (2022). A method for diagnosing depression: Facial expression mimicry is evaluated by facial expression recognition. J. Affect. Disord..

[5] Skinner E.A., Zimmer-Gembeck M.J., Connell J.P., Eccles J.S., Wellborn J.G. (1998). Individual differences and the development of perceived control. Monogr. Soc. Res. Child Dev..

[6] Jeong M., Ko B.C. (2018). Driver’s facial expression recognition in real-time for safe driving. Sensors.

[7] Whitehill J., Serpell Z., Lin Y.-C., Foster A., Movellan J.R. (2014). Engaged Faces: Automatic Recognition of Student Engagement from Facial Expressions. IEEE Trans. Affect. Computing..

[8] Abedi A., Khan S.S. (2024). Affect-driven ordinal engagement measurement from video. Multimedia Tools Appl..

[9] Dhall A., Goecke R., Lucey S., Gedeon T. (2011). Static facial expression analysis in tough conditions: Data, evaluation protocol and benchmark. Proceedings of the 2011 IEEE International Conference on Computer Vision Workshops (ICCV Workshops).

[10] Goodfellow I.J., Erhan D., Carrier P.L., Courville A., Mirza M., Hamner B., Cukierski W., Tang Y., Thaler D., Lee D.H. (2013). Challenges in representation learning: A report on three machine learning contests. Proceedings of the Neural Information Processing: 20th International Conference, ICONIP 2013.

[11] Mollahosseini A., Hasani B., Mahoor M.H. (2017). Affectnet: A database for facial expression, valence, and arousal computing in the wild. IEEE Trans. Affect. Comput..

[12] Li S., Deng W., Du J. Reliable crowdsourcing and deep locality-preserving learning for expression recognition in the wild. Proceedings of the IEEE Conference on Computer Vision and Pattern Recognition.

[13] Dhall A., Singh M., Goecke R., Gedeon T., Zeng D., Wang Y., Ikeda K. Emotiw 2023: Emotion recognition in the wild challenge. Proceedings of the 25th International Conference on Multimodal Interaction.

[14] Singh M., Hoque X., Zeng D., Wang Y., Ikeda K., Dhall A. Do i have your attention: A large scale engagement prediction dataset and baselines. Proceedings of the 25th International Conference on Multimodal Interaction.

[15] Liu S., Xu Y., Wan T., Kui X. (2023). A dual-branch adaptive distribution fusion framework for real-world facial expression recognition. Proceedings of the ICASSP 2023–2023 IEEE International Conference on Acoustics, Speech and Signal Processing (ICASSP).

[16] He K., Zhang X., Ren S., Sun J. Deep residual learning for image recognition. Proceedings of the IEEE Conference on Computer Vision and Pattern Recognition.

[17] Guo Y., Zhang L., Hu Y., He X., Gao J. (2016). Ms-celeb-1m: A dataset and benchmark for large-scale face recognition. Proceedings of the Computer Vision–ECCV 2016: 14th European Conference.

[18] Zhang X., Li J., Hua Z. (2022). MRSE-Net: Multiscale residuals and SE-attention network for water body segmentation from satellite images. IEEE J. Sel. Top. Appl. Earth Obs. Remote Sens..

[19] Song J., He M., Feng J., Shen B. (2024). Bridging the Gaps: Utilizing Unlabeled Face Recognition Datasets to Boost Semi-Supervised Facial Expression Recognition. arXiv.

[20] Fei Z., Zhang B., Zhou W., Li X., Zhang Y., Fei M. (2025). Global multi-scale extraction and local mixed multi-head attention for facial expression recognition in the wild. Neurocomputing.

[21] Xu J., Li Y., Yang G., He L., Luo K. (2024). Multiscale facial expression recognition based on dynamic global and static local attention. IEEE Trans. Affect. Comput..

[22] Savchenko A.V., Savchenko L.V., Makarov I. (2022). Classifying emotions and engagement in online learning based on a single facial expression recognition neural network. IEEE Trans. Affect. Comput..

[23] Zheng C., Mendieta M., Chen C. Poster: A pyramid cross-fusion transformer network for facial expression recognition. Proceedings of the IEEE/CVF International Conference on Computer Vision.

[24] Hsu W.Y., Chiang T.H. (2024). Triple-Attribute Perceptron Facial Expression Recognition in Real-World Environments. IEEE Trans. Consum. Electron..

[25] LeCun Y., Boser B., Denker J., Henderson D., Howard R., Hubbard W., Jackel L. (1989). Handwritten digit recognition with a back-propagation network. Adv. Neural Inf. Process. Syst..

[26] Krizhevsky A., Sutskever I., Hinton G.E. (2012). Imagenet classification with deep convolutional neural networks. Adv. Neural Inf. Process. Syst..

[27] Wang L., Guo S., Huang W., Qiao Y. (2015). Places205-vggnet models for scene recognition. arXiv.

[28] Targ S., Almeida D., Lyman K. (2016). Resnet in resnet: Generalizing residual architectures. arXiv.

[29] Arnab A., Dehghani M., Heigold G., Sun C., Lučić M., Schmid C. Vivit: A video vision transformer. Proceedings of the IEEE/CVF International Conference on Computer Vision.

[30] Woo S., Park J., Lee J.Y., Kweon I.S. Cbam: Convolutional block attention module. Proceedings of the European Conference on Computer Vision (ECCV).

[31] Ashish V. (2017). Attention is all you need. Adv. Neural Inf. Process. Syst..

[32] Xue F., Wang Q., Tan Z., Ma Z., Guo G. (2022). Vision transformer with attentive pooling for robust facial expression recognition. IEEE Trans. Affect. Comput..

[33] Ma F., Sun B., Li S. (2021). Facial expression recognition with visual transformers and attentional selective fusion. IEEE Trans. Affect. Comput..

[34] Liu Y., Zhang X., Kauttonen J., Zhao G. (2023). Uncertain facial expression recognition via multi-task assisted correction. IEEE Trans. Multimedia.

[35] Xue F., Wang Q., Guo G. Transfer: Learning relation-aware facial expression representations with transformers. Proceedings of the IEEE/CVF International Conference on Computer Vision.

[36] Mao J., Xu R., Yin X., Chang Y., Nie B., Huang A., Wang Y. (2024). Poster++: A simpler and stronger facial expression recognition network. Pattern Recognit..

[37] Wang K., Peng X., Yang J., Lu S., Qiao Y. Suppressing uncertainties for large-scale facial expression recognition. Proceedings of the IEEE/CVF Conference on Computer Vision and Pattern Recognition.

[38] Farzaneh A.H., Qi X. Facial expression recognition in the wild via deep attentive center loss. Proceedings of the IEEE/CVF Winter Conference on Applications of Computer Vision.

[39] Zhao Z., Liu Q., Zhou F. (2021). Robust lightweight facial expression recognition network with label distribution training. Proc. AAAI Conf. Artif. Intell..

[40] Zhao Z., Liu Q., Wang S. (2021). Learning deep global multi-scale and local attention features for facial expression recognition in the wild. IEEE Trans. Image Process..

[41] Chen S., Wang J., Chen Y., Shi Z., Geng X., Rui Y. Label distribution learning on auxiliary label space graphs for facial expression recognition. Proceedings of the IEEE/CVF Conference on Computer Vision and Pattern Recognition.

[42] Wang K., Peng X., Yang J., Meng D., Qiao Y. (2020). Region attention networks for pose and occlusion robust facial expression recognition. IEEE Trans. Image Process..

[43] Suharto E., Widodo A.P., Sarwoko E.A. (2020). The use of mobilenet v1 for identifying various types of freshwater fish. J. Phys. Conf. Ser..

[44] Tadepalli Y., Kollati M., Kuraparthi S., Kora P. (2021). EfficientNet-B0 Based Monocular Dense-Depth Map Estimation. Trait. Signal.

[45] Li H., Wang N., Ding X., Yang X., Gao X. (2021). Adaptively learning facial expression representation via cf labels and distillation. IEEE Trans. Image Process..

[46] Yang L., Yu H., Cheng Y., Mei S., Duan Y., Li D., Chen Y. (2021). A dual attention network based on efficientNet-B2 for short-term fish school feeding behavior analysis in aquaculture. Comput. Electron. Agric..

[47] Cai J., Meng Z., Khan A.S., Li Z., O’Reilly J., Tong Y. (2022). Probabilistic attribute tree structured convolutional neural networks for facial expression recognition in the wild. IEEE Trans. Affect. Comput..

[48] Jiang J., Deng W. (2022). Disentangling identity and pose for facial expression recognition. IEEE Trans. Affect. Comput..

[49] Wang Q., Wu B., Zhu P., Li P., Zuo W., Hu Q. ECA-Net: Efficient channel attention for deep convolutional neural networks. Proceedings of the IEEE/CVF Conference on Computer Vision and Pattern Recognition.

